# Nrf2/HO-1 mediates the neuroprotective effect of mangiferin on early brain injury after subarachnoid hemorrhage by attenuating mitochondria-related apoptosis and neuroinflammation

**DOI:** 10.1038/s41598-017-12160-6

**Published:** 2017-09-19

**Authors:** Zefeng Wang, Songxue Guo, Junxing Wang, Yuanyuan Shen, Jianmin Zhang, Qun Wu

**Affiliations:** 1 Department of Neurosurgery, Second Affiliated Hospital, Zhejiang University, College of Medicine, 88 Jiefang Road, Hangzhou, 310009 Zhejiang China; 2 Department of Plastic surgery, Second Affiliated Hospital, Zhejiang University, College of Medicine, 1511 Jianghong Road, Hangzhou, 310009 Zhejiang China; 3 Department of Neuroscience care unit, Second Affiliated Hospital, Zhejiang University, College of Medicine, 88 Jiefang Road, Hangzhou, 310009 Zhejiang China

## Abstract

Early brain injury (EBI) is involved in the process of cerebral tissue damage caused by subarachnoid hemorrhage (SAH), and multiple mechanisms, such as apoptosis and inflammation, participate in its development. Mangiferin (MF), a natural C-glucoside xanthone, has been reported to exert beneficial effects against several types of organ injury by influencing various biological progresses. The current study aimed to investigate the potential of MF to protect against EBI following SAH via histological and biological assessments. A rat perforation model of SAH was established, and MF was subsequently administered via intraperitoneal injection at a low and a high dose. High-dose MF significantly lowered the mortality of SAH animals and ameliorated their neurological deficits and brain edema. MF also dose-relatedly attenuated SAH-induced oxidative stress and decreased cortical cell apoptosis by influencing mitochondria-apoptotic proteins. In addition, MF downregulated the activation of the NLRP3 inflammasome and NF-*κ*B as well as the production of inflammatory cytokines, and the expression of Nrf2 and HO-1 was upregulated by MF. The abovementioned findings indicate that MF is neuroprotective against EBI after SAH and Nrf2/HO-1 cascade may play a key role in mediating its effect through regulation of the mitochondrial apoptosis pathway and activation of the NLRP3 inflammasome and NF-*κ*B.

## Introduction

Subarachnoid hemorrhage (SAH), a severe subtype of stroke, brings high morbidity and mortality to patients suffering from ruptured aneurysms and other cerebrovascular emergencies^1,2^. Early brain injury (EBI), which usually occurs within 72 h after SAH, has been reported to play a key role in determining the prognosis of SAH patients^3–5^. In clinic, most of current therapeutic methods focus on the treatment of vascular events, including cerebro-spinal fluid (CSF) drainage, antifibrinolytic agents, coagulant drugs, removal of pathogeny, etc., however, all above intervention remain limited. The failure of clinical treatment to reduce cerebral vasospasm incidence results in researchers’ attention to EBI, which is considered as an important therapeutic target^6^. So far, there is no specific medicine or intervention determined for EBI, and there is still a space for researchers to explore new drugs or agents to improve therapeutic effect. Multiple pathophysiological mechanisms have been identified as being involved in the development of EBI secondary to SAH, including oxidative stress, neural cell apoptosis, and neuroinflammation^3,7^. Current studies of prevention and therapy also mainly focus on these mechanisms.

Mangiferin (MF), usually isolated from a variety of natural plants such as mango trees, *Iris unguicularis*, *Anemarrhena asphodeloides* and the family *Gentianaceae*, was initially identified in the late 1960s as a type of xanthone derivative and C-glucosyl xanthone (2C-${\rm{\beta }}$-D-glucopyranosyl-1,3,6,7-tetrahydroxyxanthone)^8,9^. Due to its special structure with C-glycosidic linkage, MF exhibits powerful antioxidant and free radical scavenging properties^10,11^. In addition, MF is considered to protect against various organ injuries arising from different causes, and it has anti-inflammatory, anti-apoptotic, and antioxidant benefits^8,12–15^. MF displays anti-diabetic, anti-cancer, and anti-infection effects by regulating inflammation, apoptosis, and cell proliferation as well as mediating immunomodulation^16–19^. In addition, it has been reported to influence the activation or expression of several signal cascades, such as NF-κB, Nrf2/HO-1, mitochondria-dependent pathways, and the NLRP3 inflammasome, and to target multiple cytokines and antioxidant enzymes, such as interleukin (IL)-6, tumor  necrosis factor (TNF), superoxide dismutase (SOD), and catalase (CAT)^10–12,17,19–21^. In terms of the central nervous system, Yang *et al*. demonstrated that MF effectively attenuated ischemic brain injury by regulating the release of inflammatory cytokines and upregulating the activity of endogenous antioxidant enzymes and the expression of the Nrf2/HO-1 signaling cascade^22^. Marquez *et al*. reported that it attenuates neuroinflammation and oxidative damage in a rat stress model and that its targets include catalase activity, lipid peroxidation, elevated levels of proinflammatory mediators and increased activation of NF-κB^23^. Given the abovementioned pharmacological and biological properties, we hypothesized a possible benefit of MF against EBI after SAH, and we were the first to explore in detail the biological mechanisms of its effect via an experimental rat SAH model.

## Results

### Survival analysis, mortality and SAH grade

**Figure 1:**
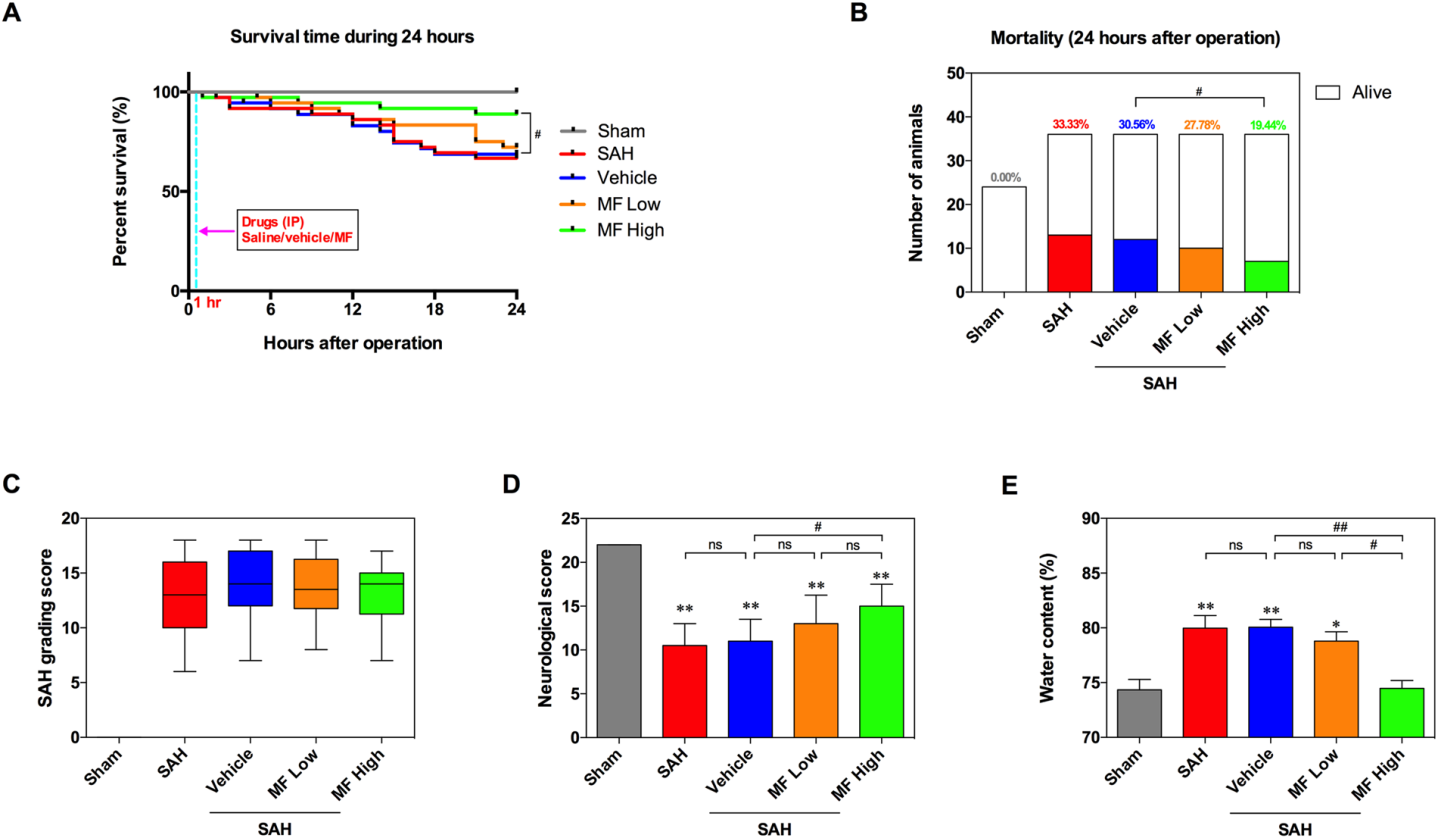
General evaluation of SAH models and the effect of MF on neurological function and brain edema in SAH rats. (**A**) Survival analysis during 24 hours after SAH. (**B**) Evaluation of Mortality at 24 hours after SAH or sham operation. (**C**) Quantification of SAH Severity. (**D**) Quantification of neurological function. (**E**) Brain water content at 24 hours after SAH or sham operation. n = 8 per group. ^#^P < 0.05, ^##^P < 0.01, ^ns^P > 0.05; *P < 0.05, **<0.01, compared with the Sham group.

Low-dose MF did not improve survival rate significantly compared with the SAH and Vehicle groups; however, high-dose MF treatment improved survival after SAH (Fig. 1A). None of the rats in the Sham group died, and there was no significant difference between the SAH and Vehicle groups (Fig. 1B). Low-dose MF treatment only gave rise to a slight and unremarkable decrease in mortality, whereas high-dose MF seemed to reduce mortality after SAH significantly (Fig. 1B). With respect to SAH grading and induction, all groups except the Sham group showed similar SAH grading scores, and there was no difference among them (Fig. 1C).

### Neurological score and water content

Compared with the sham group, both the SAH and Vehicle groups presented remarkably lower neurological scores after SAH, although there was no significant difference between the two groups (Fig. 1D). In terms of MF treatment, the low-dose group only showed a slight improvement on neurological score after SAH, whereas the high dose induced a significant increase in neurological score (Fig. 1D). The extent of brain edema was evaluated by measurement of water content. According to our observation, SAH induced a significant elevation of water content in the cerebral samples of the SAH group, and the Vehicle group showed a similar level of water content to the SAH group (Fig. 1E). Although the water content of the MF Low group decreased slightly compared with that of the Vehicle group, it was still markedly higher than that of the Sham group (Fig. 1E). However, high-dose MF treatment decreased the SAH-induced elevation of water content significantly and brought the water content close to the normal level in the Sham group (Fig. 1E).

### Oxidative stress assessment

**Figure 2:**
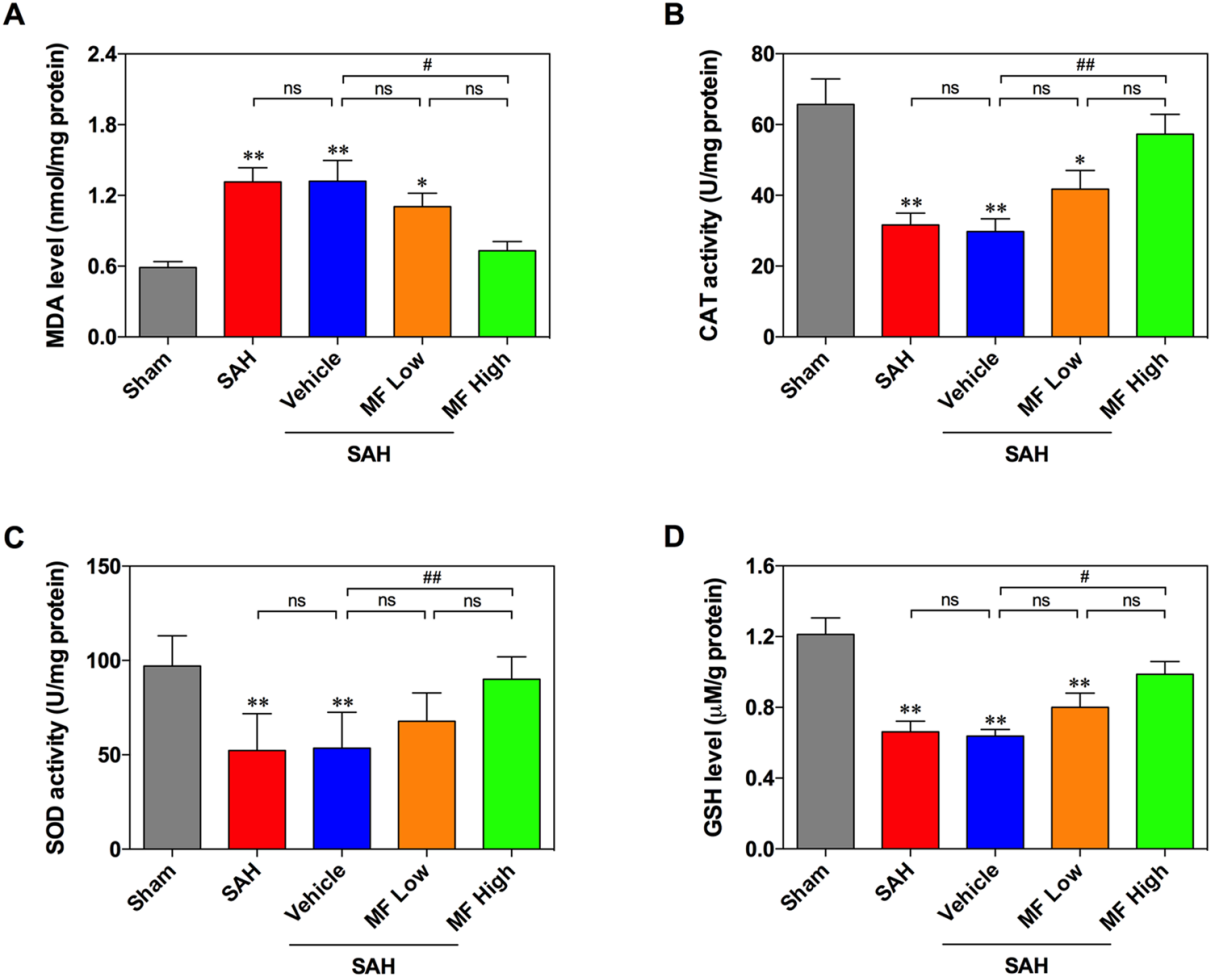
MF attenuates SAH-induced aggravation of oxidative stress in brain tissue. (**A**) Detection of MDA levels in the brain tissue of rats after SAH. (**B** and **C**) Assessment of the activities of SOD and CAT. (**D**) The detection of brain GSH. n = 8 per group, ^#^P < 0.05, ^##^P < 0.01, ^ns^P > 0.05; *P < 0.05, **<0.01, compared with the Sham group.

SAH induced a significant elevation in brain tissue MDA levels and decreased the levels of total SOD and CAT remarkably (Fig. 2). However, vehicle treatment did not attenuate SAH-induced MDA elevation and reduction of total SOD and CAT levels (Fig. 2). In terms of MF treatment, low-dose MF exhibited a weak tendency to reduce the increased level of MDA after SAH, whereas high-dose MF could significantly lower the MDA level compared with that of the Vehicle group, and its level was similar to that of the Sham group (Fig. 2A). Meanwhile, although low-dose MF seemed slightly to restore the activity of total SOD and CAT in SAH-insulted brain tissue, the effect of high-dose MF was more remarkable, bringing about a significant improvement in the activity of total SOD and CAT after SAH (Fig. 2B and C). In terms of glutathione (GSH), we observed significant decrease of GSH level in brain tissue after SAH, and a similar change was also observed in the vehicle group (Fig. 2D). High-dose MF increased GSH level significantly compared with that of the vehicle group (Fig. 2D).

### Influence of MF on basal cortical cell apoptosis after SAH

**Figure 3:**
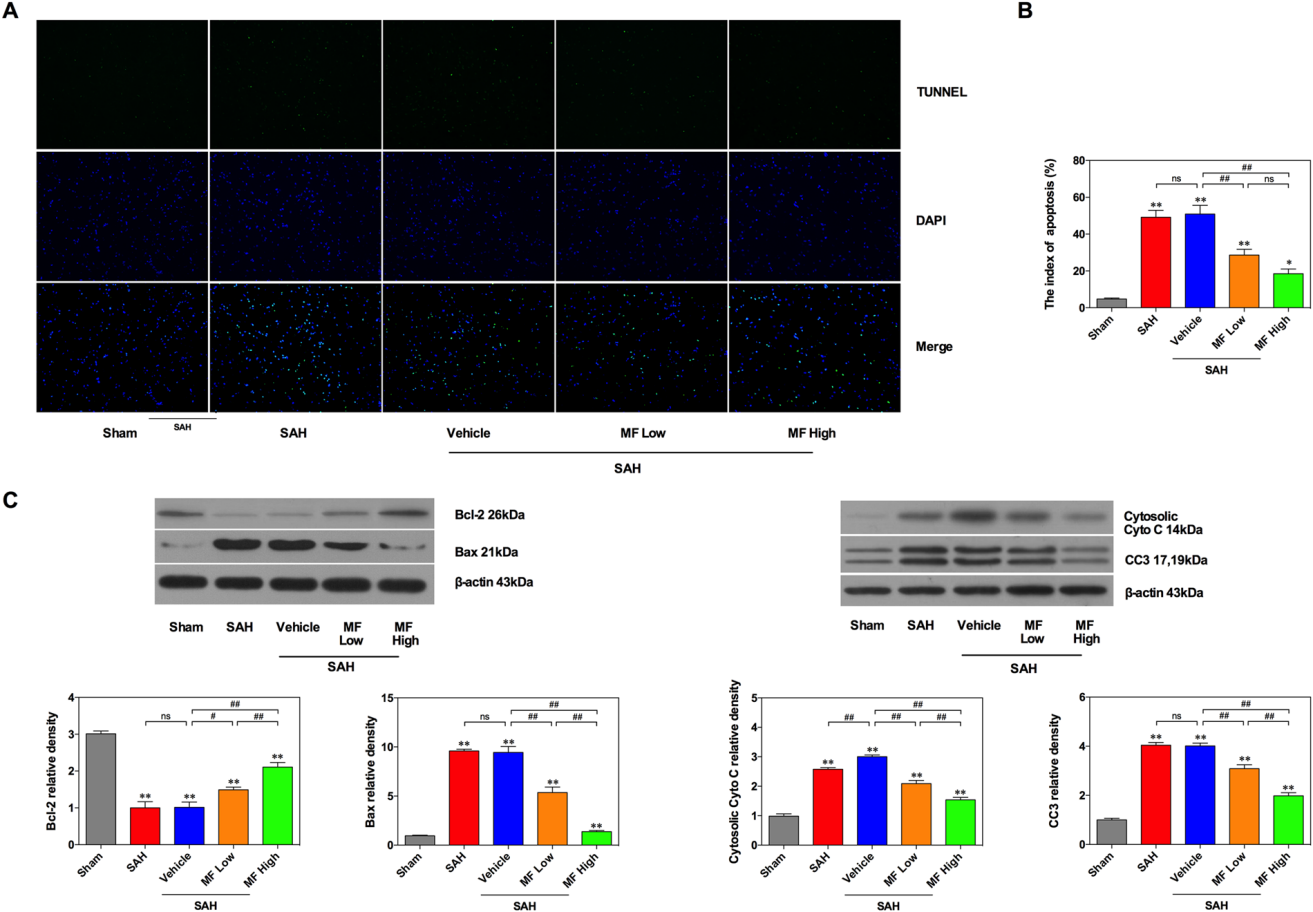
MF decreases cortical cell apoptosis in SAH rats via mitochondria-related apoptotic signals. (**A**) Representative images of TUNEL staining. (**B**) The index of Apoptosis. n = 12 per group. (**C**) Western blot analysis of the expression of Bcl-2, Bax,﻿ cytosolic cytochrome c (Cyto C) and cleaved caspase-3 (CC3). n = 6 per group. ^#^P < 0.05, ^##^P < 0.01, ^ns^P > 0.05; *P < 0.05, **<0.01, compared with the Sham group.

Cell apoptosis in brain tissue contributes to early brain injury after SAH. The TUNEL staining directly reflected the occurrence of neural cell apoptosis in the basal cortex and the distribution of apoptotic cells. After SAH, we observed an increased distribution of apoptotic cells in the basal cortex of the rat model (Fig. 3A), and the results of the apoptotic index also demonstrated an increased number of apoptotic cells after SAH compared with the sham group (Fig. 3B). A similar change could be found in the Vehicle group (Fig. 3A and B). MF treatment seemed to be able to mitigate the increased number of apoptotic cells in the brain tissue of SAH rats.

It has been reported that mitochondria-related apoptosis plays a highly important role in the regulation of neural cell apoptosis following SAH. In the mitochondria-related apoptotic pathway, Bcl-2, a pro-survival protein, regulates the release of cytochrome c (Cyto C) by influencing the permeability of the mitochondrial membrane, ultimately decreasing the cleavage and activation of apoptosis-related proteins, such as caspases. Bax, another member of Bcl-2 family, could combine with Bcl-2 to format dimer, which usually exhibit a pro-apoptotic property. The ratio of Bcl-2 to Bax suggests pro-apoptotic or anti- apoptotic regulation tendency. In this study, the western blot results showed that the protein expression of Bcl-2 in brain tissue decreased significantly after SAH, while the Bax expression revealed a significant upregulation (Fig. 3C). Furthermore, there was an obvious elevation of the protein levels of Cyto C and cleaved caspase-3 (CC3) after SAH (Fig. 3C). In addition, although vehicle administration contributed to a greater upregulation of Cyto C compared with the SAH group, it only slightly changed the expression of Bcl-2, Bax and CC3 (Fig. 3C). In term of MF treatment, even a low dose could significantly reverse the effect of SAH on the expression of the Bcl-2/Bax/Cyto C/CC3 cascade (Fig. 3C). Furthermore, compared with the low-dose group, the high-dose MF treatment triggered an enhanced effect upregulating Bcl-2 expression and downregulating Bax expression, the release of Cyto C and the cleavage of caspase 3 (Fig. 3C).

### The effect of MF on the activation of the NLRP3 inflammasome

**Figure 4:**
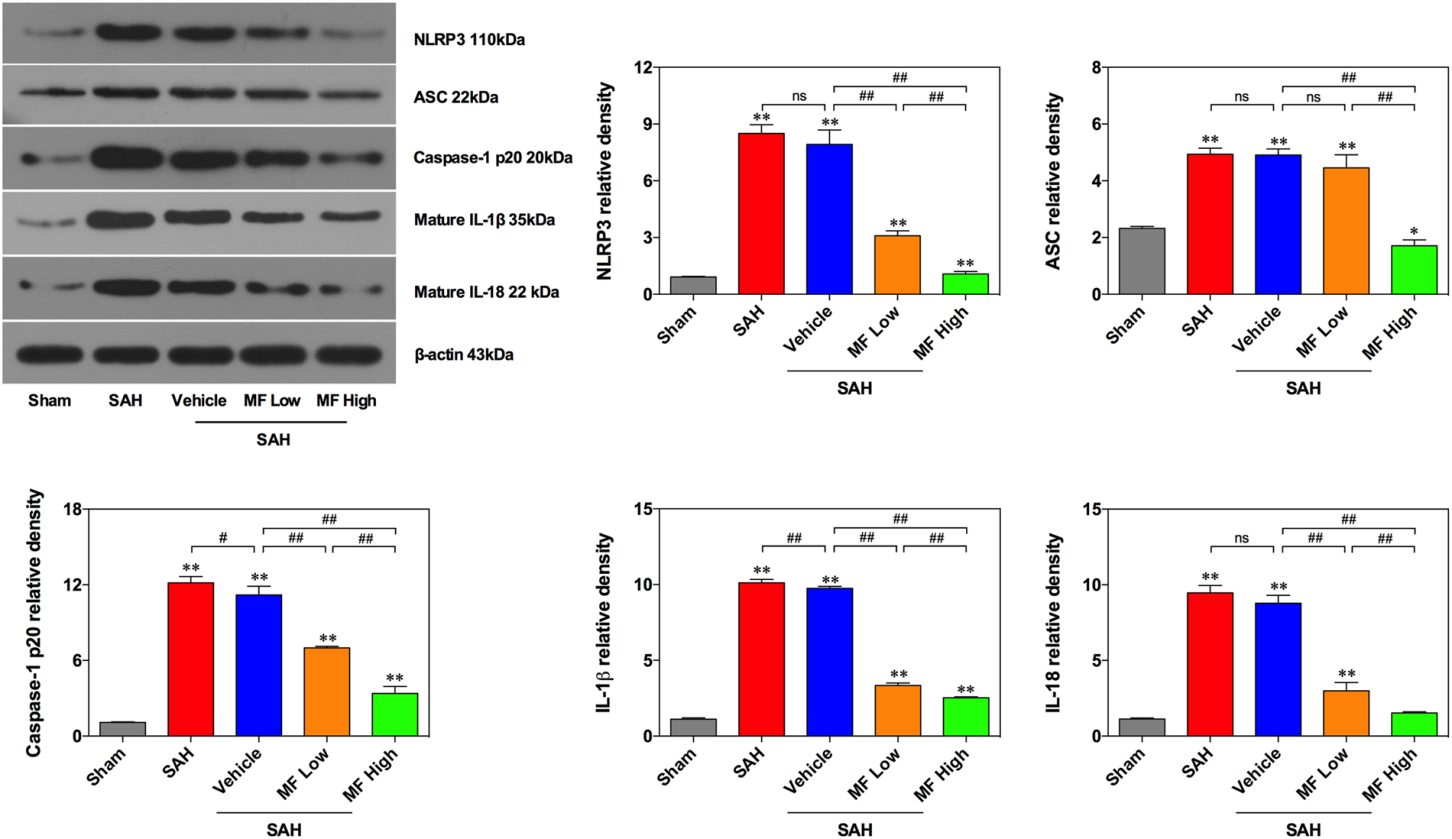
MF inhibits the activation of inflammasomes in rat brain tissues following SAH. Western blot analysis of the expression of NLRP3, ASC, and cleaved caspase-1 (p20) and the maturation of IL-1 ${\rm{\beta }}$ and IL-18 after SAH induction or MF treatment. n = 6 per group, ^#^P < 0.05, ^##^P < 0.01, ^ns^P > 0.05; *P < 0.05, **<0.01, compared with the Sham group.

The NLRP3 inflammasome has been reported to be involved in the process of neuroinflammation secondary to SAH injury. As shown in Fig. 4, after SAH insults, there was a significant upregulation of the expression of NLRP3 protein in brain tissue compared with that of the Sham group, and a similar change could also be observed in the vehicle group. Subsequently, SAH stimulation also brought about a remarkable elevation of the expression of downstream proteins, such as ASC, cleaved caspase-1 (p20), IL-1 ${\rm{\beta }}$ and IL-18, in the SAH and Vehicle groups. In both MF treatment groups, the protein expression of NLRP3 in rat brains exhibited a notable decline compared with the expression level in the Vehicle group. Correspondingly, the expression levels of ASC, cleaved caspase-1 (p20), IL-1 ${\rm{\beta }}$ and IL-18 were also downregulated significantly with MF application. Moreover, high-dose MF illustrated a stronger regulatory effects on the expression of NLRP3 and its related proteins.

### MF treatment ameliorated NF-κB-related release of inflammatory cytokines in rat brains after SAH

**Figure 5:**
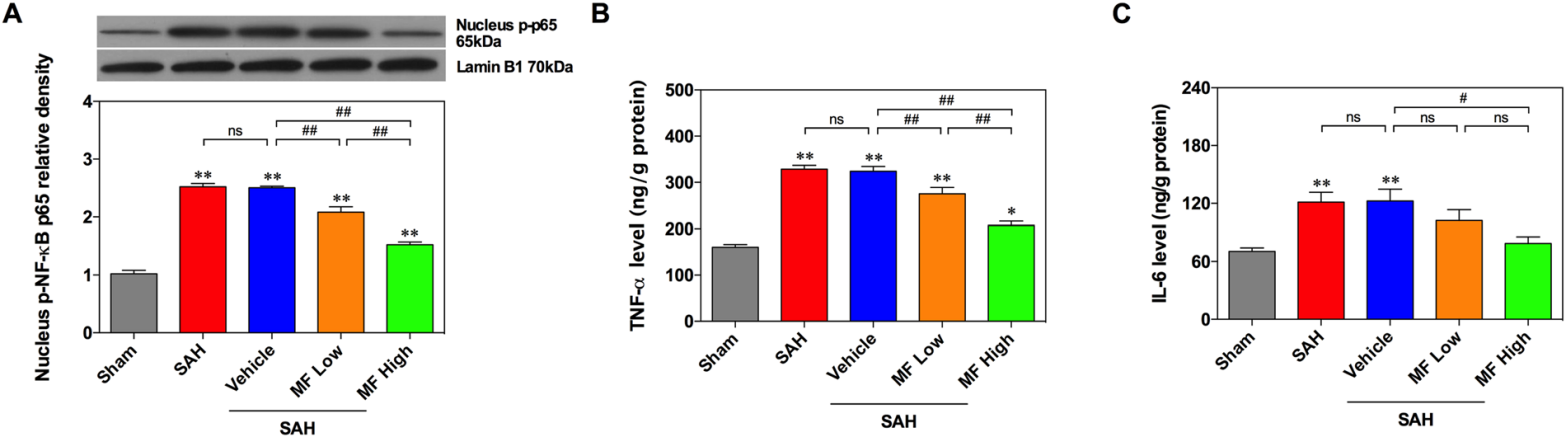
MF decreases the SAH-induced release of proinflammatory cytokines and activation of NF-κB. (**A**) Western blot assay of the phosphorylation of NF-κB p65. (**B** and **C**) ELISA assessment of TNF-${\rm{\alpha }}$ and IL-6. n = 6 per group, ^#^P < 0.05, ^##^P < 0.01, ^ns^P > 0.05; *P < 0.05, **<0.01, compared with the Sham group.

As a member of the NF-κB family, the dissociation and subsequent phosphorylation of the p65 subunit reflect the activation and translocation of the NF-κB signal (Fig. 5A). In this study, we observed similar increases in NF-κB p65 phosphorylation in both the SAH and Vehicle groups, and both of them were significantly different from the Sham group (Fig. 5A). In addition, the tissue levels of TNF-${\rm{\alpha }}$ and IL-6 increased correspondingly after SAH, according to the results of ELISA (Fig. 5B and C). Both low- and high-dose MF decreased the phosphorylation of NF-κB p65 significantly compared with the SAH and Vehicle groups, and a dose-related effect could be observed (Fig. 5A). In terms of TNF-${\rm{\alpha }}$ and IL-6, high-dose MF markedly reduced their release in the brain tissue after SAH, while low-dose MF attenuated the elevation of tissue TNF-${\rm{\alpha }}$ levels remarkably despite only slightly ameliorating IL-6 protein levels (Fig. 5B and C).

### MF treatment upregulated the cortical Nrf2/HO-1 signaling cascade in a dose-dependent manner

**Figure 6:**
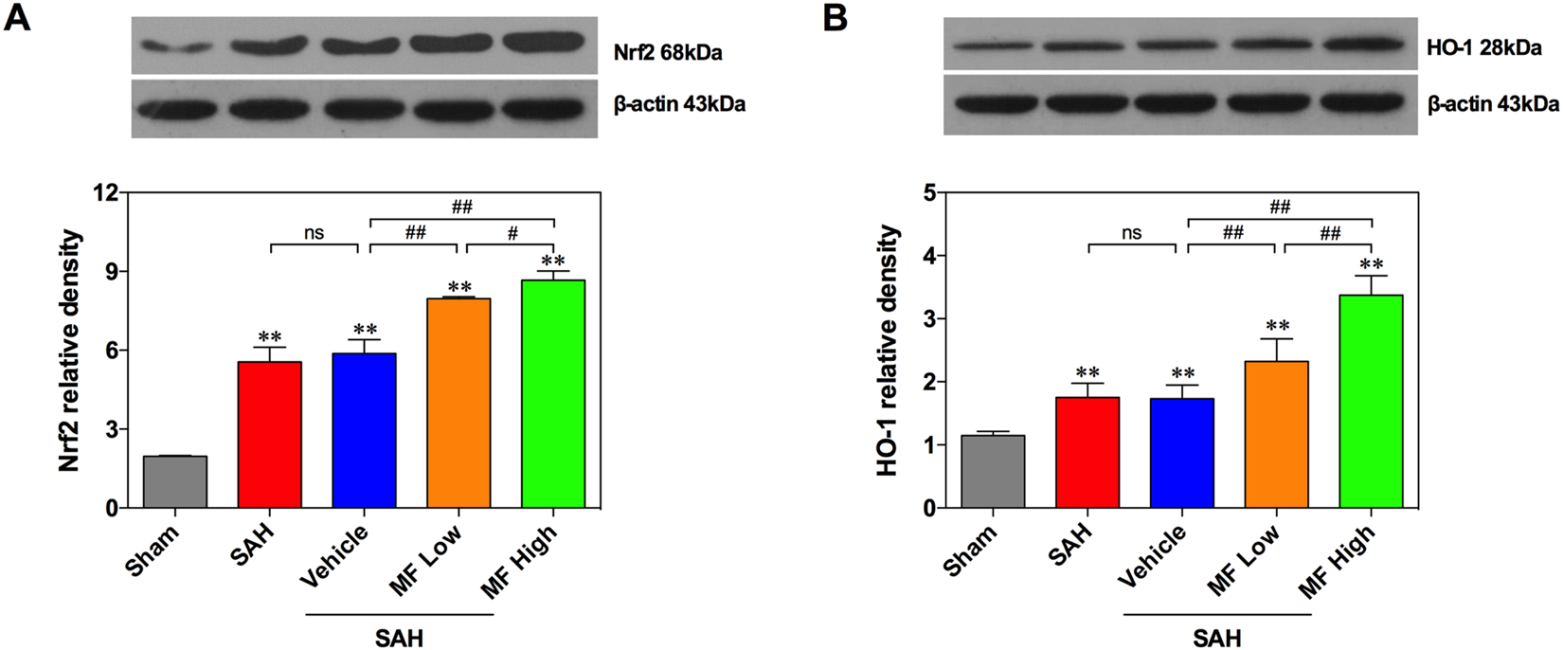
MF upregulated the expression of the Nrf2/HO-1 axis in the brains of SAH rats. Western blot assay of the expression of Nrf2 (**A**) and HO-1 (**B**) in the left hemispheres of SHA rats. n = 6 per group, ^#^P < 0.05, ^##^P < 0.01, ^ns^P > 0.05; *P < 0.05, **<0.01, compared with the Sham group.

In tandem with the changes of oxidative stress in the rat brain, SAH induced a significant downregulation of the expression of Nrf2 as well as that of its downstream protein HO-1 (Fig. 6). Vehicle treatment showed no effect on the expression of Nrf2 and HO-1 (Fig. 6). Nevertheless, both selected doses of MF could markedly upregulate the Nrf2/HO-1 signaling cascade, and the effect of the high dose was greater (Fig. 6).

## Discussion

As a primary cause for the poor outcome of SAH patients, EBI has been deemed a therapeutic target by multiple clinicians and researchers^4,7^. The term “EBI” is defined as a global brain injury that occurs in the early stage of SAH (within first 72 h)^24^. In contrast to other injurious mechanism such as vasospasm, EBI can bring about immediate damage to brain tissue after SAH^25^. Considering that the peak of cerebral vasospasm occurs at 48 h after SAH, the time point of 24 h after the insult is suggested as a suitable focus for research^26^. A series of pathophysiological changes occur during the period of EBI, such as raised intracerebral pressure, reduced cerebral blood flow (CBF), blood-brain barrier (BBB) disruption, brain swelling/edema, acute vasospasm and dysfunction of autoregulation^3^. All of these changes could result in a complex condition with global ischemia, altered ionic homeostasis, degradation of vascular integrity and excitotoxicity, which would induce the mobilization of several molecular mechanisms, such as reactive oxygen species (ROS)-mediated oxidative stress, neuroinflammation, cell death, etc.^4,25,27^. In previous studies, MF has been demonstrated as a potential protectant to prevent or alleviate several secondary organ injuries, including cerebral ischemia-reperfusion injury, inflammatory brain injury, acute liver injury, acute kidney injury, and others, and its effect seems to be achieved via various mechanisms such as anti-oxidation, anti-inflammation, and anti-apoptosis^10,11,13,15,22,28,29^. In the present study, we were the first to identify the protective effect of MF against EBI after SAH in a classic rat model. The data obtained reveal that (1) MF improves neurological deficit and tissue edema after SAH; (2) MF attenuates SAH-induced oxidative stress by decreasing lipid peroxidation, restoring endogenous enzymes (SOD and CAT) and increasing GSH; (3) MF decreases neural cell apoptosis and the activation of the mitochondrial apoptosis pathway in brain tissue after SAH; (4) In conjunction with relieving the local release of proinflammatory cytokines after SAH, MF downregulates the expression of NLRP3, ASC and cleaved caspase-1 as well as the activation of NF-κB; (5) MF treatment also effectively upregulates the expression of Nrf2 and HO-1 in the brains of SAH rats in a possible dose-related manner.

Although there was no difference observed in SAH grade among the four SAH groups, high-dose MF seemed to be able to increase the 24-h survival of SAH rats based on the analysis of survival time and mortality. Meanwhile, the assessment of neurological score, which is a direct index for the severity of EBI, also suggests a benefit of MF in attenuating early deterioration of neurological function induced by SAH. Similarly, Yang *et al*. reported a dose-dependent effect of MF on alleviating neurological deficits caused by cerebral ischemia-reperfusion in rats^22^. In some *in vivo* experiments, MF also exhibited an effective ability to ameliorate drug-induced cognitive dysfunction and anxiety behavior^28,30^. In addition, high-dose MF effectively decreases the water content of the brain in SAH rats, indicating a relief of brain edema, and this effect resembled the dose-dependent result found in a cerebral ischemia-reperfusion model^22^. All these provide a preliminary suggestion about the protective effect of MF against EBI after SAH.

Reactive oxygen species (ROS) contribute to multiple pathophysiological processes involving organ injuries and biological metabolism^31,32^. Their inappropriate overproduction can disrupt the balance between oxidation and anti-oxidation in biological organisms, ultimately triggering oxidative stress. Apart from direct oxidative damage, ROS also participate in the regulation of several molecular processes, such as inflammation and apoptosis, by influencing gene expression and the structure and function of mitochondria^33,34^. As a representative result of ROS action, lipid peroxidation can be found in multiple types of cell and tissue damage, and its end product MDA is widely used as an indicator reflecting ROS-mediated oxidative stress. In addition, the imbalance of redox status caused by ROS brings about an excessive consumption of endogenous anti-oxidant defense system, including anti-oxidant (such as GSH, etc.) or anti-oxidant enzymes (such as SOD, CAT, etc.), whose levels or activity levels can serve as indirect indices of oxidative stress. According to data from previous studies, increased lipid peroxidation and decreased activity levels of GSH and endogenous antioxidant enzymes in brain tissue can be observed following SAH attack, validating the important role of oxidative stress in early EBI^1,2,35,36^. Most of the reported possible interventions to protect against EBI after SAH rely on the behavior of antioxidant substance or enzymes to eliminate or relieve ROS-induced oxidative stress^1,37–40^. With respect to MF, results from *in vivo* and *in vitro* experiments have displayed its effectiveness against oxidative stress^8,41^. On one hand, MF has a xanthonoid structure with C-glucosyl linkage along with the presence of multiple -OH groups, enabling it to neutralize many free radicals^42^. On the other hand, MF is able to reduce the production of ROS by preventing Fenton-type reactions and to attenuate corresponding lipid peroxidation reactions^42,43^. The Nrf2/HO-1 cascade has also been suggested to be involved in the antioxidant properties of MF in light of multiple lines of experimental evidence^20,22,28,44^. In this study, we observed that MF could reduce SAH-induced elevation of MDA levels in the rat brain and that the higher dose was more effective. Meanwhile, the investigation of anti-oxidant defense system also indicated the ability of MF to improve the levels of GSH, and the activities of SOD and CAT in SAH-afflicted rat brains. Both results demonstrated that MF could attenuate increased oxidative stress in rat brain tissue following SAH by regulating lipid peroxidation and endogenous antioxidant defense. Moreover, an upregulation of Nrf2 and HO-1 protein expression in SAH rats was detected by western blotting after MF administration, indicating the possible involvement of Nrf2/HO-1 in the anti-oxidant activity of MF as mentioned in other studies. In addition to its classic role in antagonizing ROS-mediated oxidative stress, the Nrf2/HO-1 axis is also associated with the regulation of several biological processes, such as apoptosis and inflammation^45,46^. A more detailed discussion will follow in our comments on the effects of MF on neural apoptosis and neuroinflammation.

As a type of energy-dependent programmed cell death, apoptosis contributes to the development of a variety of cell and tissue injuries mediated by diverse causes. In the central nervous system (CNS) specifically, it also plays an important role in the pathophysiological progression of ischemia and stroke-induced brain or spinal injury, inflammatory brain injury, drug toxicity-induced brain or spinal injury and other conditions; therefore, interventions targeting apoptosis seem to be a reasonable way to protect against CNS damage^3^. In terms of SAH, Zubkov *et al*. first identified the typical endothelial call apoptosis secondary to SAH-induced cerebral vasospasm^47^. Furthermore, apoptosis was also detected in cortical, subcortical or hippocampal neurons, along with corresponding changes in intrinsic and extrinsic apoptotic signaling pathways. The TUNEL staining result in the present study revealed that there was a significant augmentation in the number of apoptotic neural cells in rat basal cortex, similar to reported results from previous studies; however, both dosages of MF could effectively decrease cortical cell apoptosis after SAH, suggesting the involvement of anti-apoptosis in the mechanism of neuroprotection of MF. Pal *et al*. investigated the effect of MF on lead [Pb(II)]-induced hepatic injury and found that MF was beneficial to attenuate lead-induced cell apoptosis in murine liver tissue via the mitochondria-dependent pathway, and a similar regulatory effect of MF was also reported in their study on diabetic nephropathy^11,48^. In SK-N-SH neuroblastoma cells, Kavitha *et al*. demonstrated that MF ameliorated rotenone-induced cell apoptosis by influencing the expression of mitochondria-related apoptotic proteins^49^. Although various signals have been suggested to take part in the regulation of neural apoptosis after SAH, the mitochondria-related apoptosis pathway is still an unignorable player in SAH-induced EBI^4,50^. Cyto C plays a key role in the activation of apoptosis-related proteins such as caspases, and its release from mitochondria is commonly manipulated by the Bcl-2 family of apoptotic proteins and by mitochondrial permeability, both of which can be impacted by ROS^50^. In this study, we observed a series of changes in the expression of the mitochondria-related apoptotic proteins Bcl-2, Bax, Cyto C and cleaved caspase-3 (CC3) in cortical tissue after SAH, in accordance with previous reports. When MF was administered, all SAH-induced changes in the expression of these proteins were reversed to a remarkable extent, suggesting the possible effectiveness of MF in regulating mitochondria-related neural apoptosis. Mitochondrium serves a crucial role in the regulation of energy generation. Mitochondrium is critical for energy generation and its functional integrity is essential for cell survival. The integrity of its function could affect the release of Cyto C and resultantly influence cell survival. S. Prabhu *et al*. observed the cardioprotective effect of MF in myocardiac infarcted rats, which was attributed to its preservation on mitochondrial function via activation of mitochondrial energy metabolism and alleviate the damaging changes found in mitochondrial ultrastructure^51^. In addition, in an *in vitro* research, Song J. *et al*. had made a systemic investigation of the detail effect of MF on mitochondrial function, and demonstrated that MF could reduce Cyto C release and further protect cell from apoptosis by inhibiting  capase-3 activation, resulting from its prevention against mitochondrial membrane potential and its restoration of mitochondrial membrane potential^52^. In the present study, we observed similar beneficial effects of MF on mitochondrial Cyto C release and related apoptosis in SAH rats, which provide us some primary evidences to deduce the mitochondrial role in MF’s neuroprotection. Combined with the above reported results from *in vivo* and *in vitro*, in SAH rats, MF’s neuroprotection may also relate to its effect on mitochondrial function, and a future study specialized in investigation referring to mitochondria will be helpful to determine the value of MF further. On the other hand, the Nrf2/HO-1 cascade has been reported to inhibit apoptosis based on its effect on mitochondria-related apoptotic proteins such as Bcl-2 and Bax; therefore, the anti-apoptotic property of MF in SAH rats is partly related to its upregulation of Nrf2 and HO-1 expression^53–55^.

Anti-inflammation is one of the widely reported biological activities of MF, and multiple researchers regard it as a basis underlying the benefits of MF against various organ injuries^10^. Both Yang *et al*. and Kasbe *et al*. suggested that the improvement of neurological function in animals with brain injury was associated with MF’s effect on inhibiting the overproduction of proinflammatory cytokines, along with its relief of oxidative stress^22,56^. Furthermore, NF-κB is identified as a crucial signal mediating the regulatory effect of MF on proinflammatory cytokines, based on studies concerning nervous system disorders, cardiac injury, lung injury, renal injury, liver disorders, and others^10,29^. In terms of SAH insults, our previous studies demonstrated the activation of NF-κB in cerebrovascular and basal cortical tissues, and protective agents seemed to decrease local production of proinflammatory cytokines via inhibition of NF-κB activation^2,57^. In addition to NF-κB signaling, the NLRP3 inflammasome has been examined as a novel target in studies concerning EBI after SAH^3^. As a protein complex, the NLRP3 inflammasome usually contains apoptosis-associated speck-like protein containing CARD (ASC), caspase-1 and other functional proteins, and its members can be sequentially activated as an immune response when facing external stimuli. The maturation of proinflammatory cytokines, such as IL-1 ${\rm{\beta }}$ and IL-18, is a main result of NLRP3 inflammasome-based promotion. Recently, He *et al*. observed that MF inhibited the activation of the NLRP3 inflammasome, which contributed to the nephroprotective effect of MF against sepsis-induced acute kidney injury along with its upregulation of Nrf2^15^. In this study, we not only found that MF could effectively decrease SAH-induced overproduction of proinflammatory cytokines (IL-1 ${\rm{\beta }}$, IL-6, IL-18, TNF-${\rm{\alpha }}$) but also detected its corresponding regulatory effects on the activation of NF-κB and the NLRP3 inflammasome. Considering the reported regulatory effect of NF-κB on the activation of the NLRP3 inflammasome, they mediate neuroinflammation cooperatively rather than in parallel^58^. Moreover, ROS is believed to act upstream of NF-κB and the NLRP3 inflammasome, which could partially explain the regulatory effect of Nrf2/HO-1 on inflammation^45^. In both *in vivo* and *in vitro* studies, researchers also observed the opposite relation between Nrf2/HO-1 and NLRP3-mediated inflammation; the introduction of an HO-1 inducer and inhibitor helped to clarify the suppressive effect of HO-1 on NLRP3-mediated inflammation activation^45,59–61^. He *et al*. and Pan *et al*. suggested that MF upregulates Nrf2 expression and inhibits the activation of the NLRP3 inflammasome based on their experiments with endotoxin-induced acute liver and kidney injuries^12,15^. The role of the Nrf2/HO-1 cascade in inhibiting oxidative stress-sensitive NF-κB activation has also been suggested in several prior studies^62–64^.

**Figure 7:**
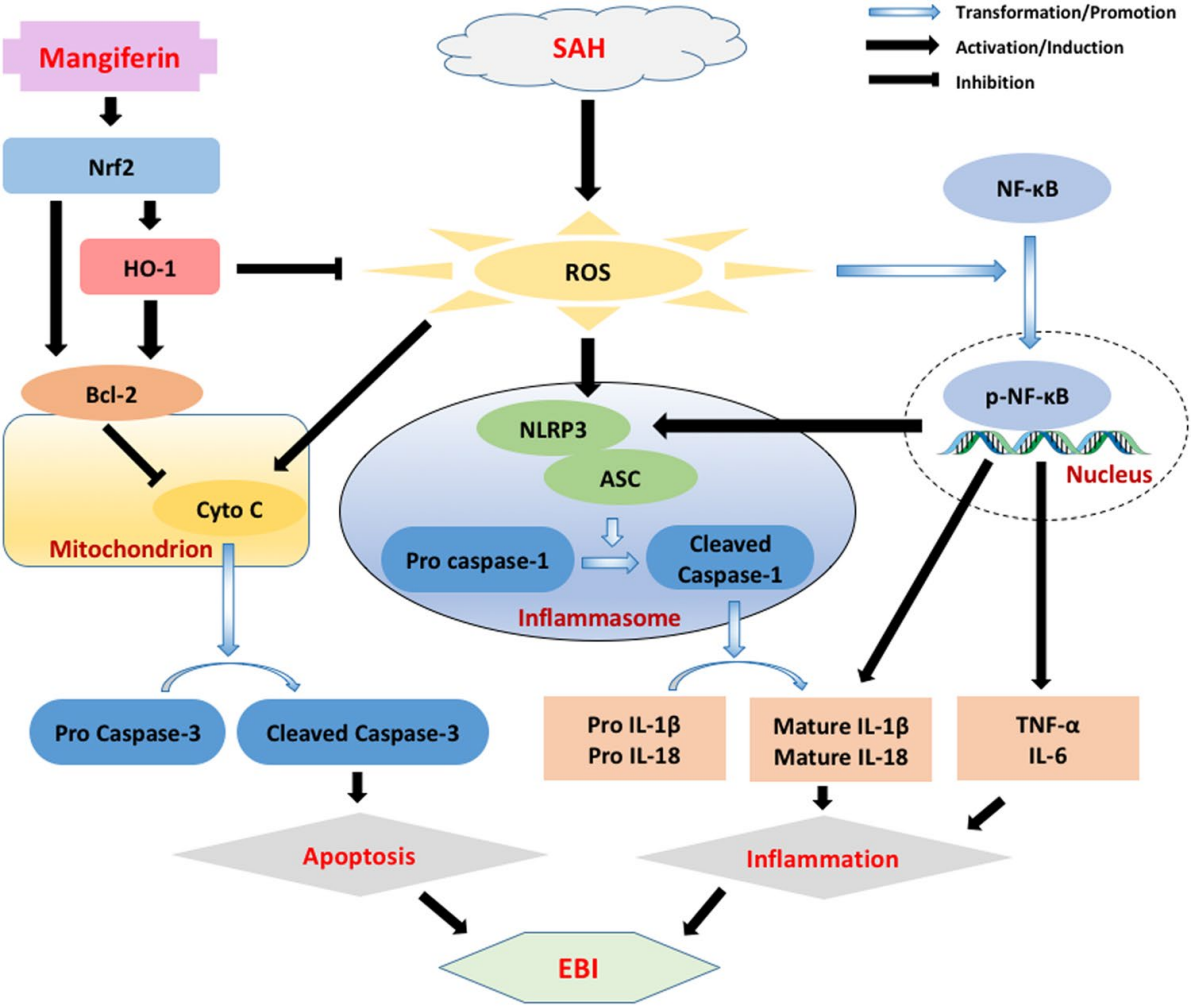
A schematic diagram of the potential mechanisms of MF’s neuroprotective against EBI after SAH.

In summary, our current study demonstrated the protective effect of MF against EBI after SAH in a rat model. The specific targets of MF, including mitochondria-related neural apoptosis and local neuroinflammation and the Nrf2/HO-1 axis, may play crucial roles in mediating the biological benefit of MF. As shown in Fig. 7, our investigation has drawn a network of potential mechanisms involved in the protection of MF; however, our study still has some limitation. First, some other pathophysiological changes, such as BBB disruption, as well as signaling pathways may also participate in the effect of MF on EBI after SAH; further assessment of this possibility is needed. In addition, specific antagonists and agonists will be useful for clarifying and validating the regulatory effect of MF on the signals examined in our study. To further evaluate the role of mitochondria in the protective effect of MF on EBI after SAH, more measurements, like mitochondrial transition pore, mitochondrial membrane potential, VDAC level, should be taken into account for clarifying the detailed changes happened in mitochondrial function and membrane stability. Although the two dosages selected in this study give a preliminary view of the dose-dependent effect of MF, more detailed classification of MF dosage is still required to establish the optimal dose and frequency of usage in future exploration.

## Methods

### Animals

This study used adult male Sprague-Dawley (SD) rats (weighing approximately 310–340 g), which were purchased from the Animal Center of Zhejiang Chinese Medical University (Hangzhou, China) and were housed in an air-filtered unit with consistent temperature and stable humidity. All animals were kept under a 12-h/12-h light/dark cycle and had free access to food and water. The study was approved by the Zhejiang University Committee on Animal Care (2016–144) and strictly abided by the National Institutes of Health Guidelines for the Care and Use of Laboratory Animals.

### Rat SAH model and sham operation

The classic SAH model was constructed by endovascular perforation as described previously^2,65^. Briefly, each rat was anesthetized with pentobarbital sodium (50 mg/kg, intraperitoneal injection), and the left carotid artery and its branches were separated and exposed. Then, the left external carotid artery was transected distally and reflected caudally in line with the internal carotid artery (ICA). A blunted 4–0 monofilament nylon suture was placed in the external carotid artery and advanced through the ICA until resistance was felt, approximately 18–20 mm from the common carotid artery bifurcation. The suture was further advanced approximately 3 mm to perforate the ICA near its intracranial bifurcation. The suture was withdrawn after 15 s. The sham operation used the same surgical procedures as the SAH model except for the perforation.

### SAH grading

The severity of the SAH in the rat model was quantified according to a published grading scale^2^. The evaluation was conducted after the animals were euthanized, and the scale was based on the amount of subarachnoid blood in six segments of the basal cistern: 0, no subarachnoid blood; 1, minimal subarachnoid blood; 2, moderate blood with visible arteries; and 3, blood clot covering all arteries within the segment. The sum of the scores in all six segments contributed to the final score, which ranged from 0 to 18.

### Experimental design and drug delivery

One hundred sixty-eight SD rats were randomly divided into five groups: the Sham group (n = 24), the SAH group (n = 36), the SAH + Vehicle group (n = 36), SAH + Mangiferin (MF) Low (n = 36), and SAH + MF High (n = 36). Animals in both the Sham and SAH groups received 0.9% saline (intraperitoneal injection (IP)) after the sham or SAH operation. The Vehicle group was treated with an equal amount of vehicle solution (0.1% dimethylsulfoxide (DMSO) in 0.9% saline, IP), which was used for dissolving MF. After SAH, the Low-dose group (MF Low) received 20 mg/kg MF (IP), while High-dose group (MF high) was treated with 100 mg/kg MF (IP). The MF was purchased from Sigma-Aldrich (06279, MO, USA), and the concentrations were determined referring to previous reports^66,67^. All animals were sacrificed at 24 h after the operation, and brain samples were dissected after cardiac perfusion with phosphate-buffered saline (PBS) (pH = 7.2). Expect several samples (n = 8) kept for the immediate measurement of brain water content, the rest of the samples were maintained in 4% paraformaldehyde at 4 °C or in a freezer at −80 °C for subsequent experiments.

### The assessment of survival, mortality and neurological function

The survival time of each animal was recorded in a 24 h-period, and the data was applied with a survival analysis. The mortality was calculated at 24 h after operation, and the number of dead rats divided by the total number of rats yielded the mortality in each group. In this study, the modified Garcia test was applied for the evaluation of neurological deficits in a blind manner as described previously. The score ranged from 3 to 22 and was based on the following tests: 1) spontaneous activity (0–3), 2) symmetry in the movement of all four limbs (0–3), 3) forepaw outstretching (0–3), 4) climbing (1–3), 5) body proprioception (1–3), 6) response to whisker stimulation (1–3), and 7) beam balance (1–4). The severer the neurological deficit, the lower the score.

### Brain water content

Rat brain edema, represented as brain water content, was assessed by a method described previously^36^. Specially, the certain amount of brains of rats (n = 8) sacrificed 24 h after the SAH or sham operations were removed in each group, and then were quickly separated into the left hemisphere, right hemisphere, and cerebellum. Left hemispheres were immediately weighed to obtain the wet weight and were then dried at 105 °C for 24 h to obtain the dry weight. The result was calculated according to the following formula: [(wet weight dry weight)/wet weight] × 100%.

### Evaluation of oxidative stress

The left basal cortical samples, which faced the blood clot, were collected at 24 h after SAH or sham operation for detection. Malondialdehyde (MDA), total superoxide dismutase (total SOD, Cu-Zn and Mn SOD), catalase (CAT) and reduced glutathione (GSH) were selected to assess the level of oxidative stress in each group. The commercial kits were purchased from KeyGen Biotech (total SOD and CAT) (Nanjing, China) and Beyotime Biotech (GSH) (Shanghai, China), and all indices were measured according to the manufacturers’ protocols.

### TUNEL staining

Five-micron-thick slices of rat brain samples were subjected to the terminal deoxynucleotidyl transferase-mediated dUTP nick-end labelling (TUNEL) staining. Briefly, after blocking with 3% H_2_O_2_ and administering 0.25% Triton X-100, we stained the slices using a commercial cell death detection kit purchased from Roche Diagnostics (Indianapolis, IN, USA). Afterward, the sections were rinsed and then mounted with VECTASHIELD HardSet Antifade Mounting Medium with DAPI (H-1500, Vector Labs, CA, USA). The slices were observed and photographed under a fluorescence microscope (DM5500B, Leica, Solms, Germany).

### Western Blotting

After being homogenized with RIPA lysis buffer (Boster, Wuhan, China) for 1 h on ice, brain lysates were centrifuged at 1000 *g* and 4 °C for 10 min to obtain the supernatants. Subsequently, equal amounts of protein sample (50 μg) mixed with loading buffer were subjected to 12% SDS-PAGE and transferred onto nitrocellulose membranes by electrophoresis; meanwhile, aliquots of the remaining samples were used to determine the protein concentration of each sample with a BCA kit (KeyGEN Biotech, Nanjing, China). The membranes (PVDF) were subsequently blocked and incubated overnight at 4 °C with the following primary antibodies: anti-p-NF-κB p65 (1:500) (ab76302, Abcam, Cambridge, UK), anti-Bcl-2 (1:1000) (3498, Cell Signaling Technology, MA, USA), anti-Bax(1:2000)(ab32503, Abcam, Cambridge, UK), anti-cytochrome c (Cyto C) (1:1000) (11940, Cell Signaling Technology, MA, USA), anti-cleaved caspase-3 (CC3) (1:1000) (9661, Cell Signaling Technology, MA, USA), anti-NLRP3 (1:1000) (15101, Cell Signaling Technology, MA, USA), anti-ASC (1:1000) (67824, Cell Signaling Technology, MA, USA), anti-caspase-1 p20 (1:1000) (ab207802, Abcam, Cambridge, UK), anti-IL-1β (1:1000) (ab2105, Abcam, Cambridge, UK), anti-IL-18 (1:1000) (ab71495, Abcam, Cambridge, UK), anti-Nrf2 (1:1000) (ab62352, Abcam, Cambridge, UK), anti-HO-1 (1:1000) (70081, Cell Signal Technology, MA, USA), anti-lamin B1 (1:2000) (ab133741, Abcam, Cambridge, UK) and anti-β-actin (1:2000) (SC-47778, Santa Cruz, CA, USA) blotted on the same membranes as a control. The bands were detected with SuperSignals West Dura Extended Duration Substrate (Pierce, MA, USA), and X-ray films and were then analyzed with the Bandscan 5.0 software and compared with β-actin or lamin B1.

### ELISA detection

The frozen samples were homogenized in homogenization buffer (0.9% saline, with a ratio of 1:9 w/v, at 4 °C) using a tissue homogenizer. The final homogenate was centrifuged at 10000 *g* and 4 °C for 15 min, and the supernatants were subsequently used for inflammatory cytokine detection with commercial kits (TNF-α and IL-6, Lianshuo Biological Technology, Shanghai, China).

### Statistical analysis

All data are presented as the mean ± standard deviation (SD). GraphPad Prism version 6 (San Diego, CA, USA) was used for the statistical analyses. A twenty-four-hour Gehan–Breslow survival analysis was applied to assess survival time. An unpaired two-tailed t-test was used for pairwise mortality comparisons between groups. A Kruskal-Wallis one-way analysis of variance (ANOVA) with Dunn’s test was used for SAH and neurological score comparisons between groups. Multiple comparisons were analyzed with one-way ANOVA followed by a Bonferroni post hoc test. A value of p < 0.05 was regarded as statistically significant.
